# Testing the role of symbols in preschool numeracy: An experimental computer-based intervention study

**DOI:** 10.1371/journal.pone.0259775

**Published:** 2021-11-15

**Authors:** Daniel C. Hyde, Yi Mou, Ilaria Berteletti, Elizabeth S. Spelke, Stanislas Dehaene, Manuela Piazza

**Affiliations:** 1 Department of Psychology, University of Illinois at Urbana-Champaign, Champaign, IL, United States of America; 2 Neuroscience Program, University of Illinois at Urbana-Champaign, Champaign, IL, United States of America; 3 Department of Psychology, Guangdong Provincial Key Laboratory of Social Cognitive Neuroscience and Mental Health, Sun Yat-sen University, Guangzhou, China; 4 Educational Neuroscience Program, Gallaudet University, Washington, D.C, United States of America; 5 Department of Psychology, Harvard University, Cambridge, MA, United States of America; 6 Cognitive Neuroimaging Unit, CEA DRF/I2BM, INSERM, NeuroSpin Center, Université Paris-Sud, Université Paris-Saclay, Gif/Yvette, France; 7 Collège de France, Paris, France; 8 Center for Mind/Brain Sciences, University of Trento, Rovereto, Italy; French National Center for Scientific Research (CNRS) & University of Lyon, FRANCE

## Abstract

Numeracy is of critical importance for scholastic success and modern-day living, but the precise mechanisms that drive its development are poorly understood. Here we used novel experimental training methods to begin to investigate the role of symbols in the development of numeracy in preschool-aged children. We assigned pre-school children in the U.S. and Italy (N = 215; Mean age = 49.15 months) to play one of five versions of a computer-based numerical comparison game for two weeks. The different versions of the game were equated on basic features of gameplay and demands but systematically varied in numerical content. Critically, some versions included non-symbolic numerical comparisons only, while others combined non-symbolic numerical comparison with symbolic aids of various types. Before and after training we assessed four components of early numeracy: counting proficiency, non-symbolic numerical comparison, one-to-one correspondence, and arithmetic set transformation. We found that overall children showed improvement in most of these components after completing these short trainings. However, children trained on numerical comparisons with symbolic aids made larger gains on assessments of one-to-one correspondence and arithmetic transformation compared to children whose training involved non-symbolic numerical comparison only. Further exploratory analyses suggested that, although there were no major differences between children trained with verbal symbols (e.g., verbal counting) and non-verbal visuo-spatial symbols (i.e., abacus counting), the gains in one-to-one correspondence may have been driven by abacus training, while the gains in non-verbal arithmetic transformations may have been driven by verbal training. These results provide initial evidence that the introduction of symbols may contribute to the emergence of numeracy by enhancing the capacity for thinking about exact equality and the numerical effects of set transformations. More broadly, this study provides an empirical basis to motivate further focused study of the processes by which children’s mastery of symbols influences children’s developing mastery of numeracy.

## Introduction

The ability to understand and perform basic operations with numbers, or numeracy, begins before most children enter school [1–3]. Understanding preschool numeracy has been a topic of interest, theoretical debate, and challenge for developmental and cognitive psychologists for many decades [see 4 for a review]. Less debated are the important translational implications for education. About 25% of the population struggles with learning mathematics, and around 6.5% of the population may fit the criteria for dyscalculia: a learning disability specific to numbers and mathematics [5, 6]. Correlations between preschool numeracy and later mathematics achievement suggest that difficulties may originate before children enter school [7, 8].

Preschool numeracy interventions show promise for promoting shorter and longer-term enhancements in children’s readiness for learning school mathematics and for lowering the risk of dyscalculia [9–15]. However, curricular interventions are necessarily varied in content, in order both to capture students’ interest and to promote generalizable knowledge [10, 11, 13–16]. Thus, previous curricula do not target specific causal mechanisms to the exclusion of others. To maximize the effectiveness of preschool interventions, we need to better understand the precise factors that promote the growth of numeracy.

Methods of experimental psychology may serve to overcome this limit, using short-term, targeted experimental interventions with focused content, evaluated by assessments and analyses that directly contrast their effectiveness [e.g., 17–20, but see 21, 22]. For example, one series of studies of this nature contrasted the effects of practicing combining and comparing dot arrays on the basis of number with comparable tasks of combining and comparing arrays based on total area or brightness. The studies found some evidence that the numerical practice led to better symbolic arithmetic performance than practice on the other two types of magnitudes [17, 18]. In another study, training preschool-aged children on non-symbolic numerical comparisons that started off easier and got harder over the course of training resulted in higher standardized preschool mathematics achievement scores compared to children who trained on the same numerical comparisons presented in the reverse order (i.e., hard to easy) [20]. In these cases, focused experimental training conditions were applied and contrasted to probe the relationship between non-symbolic approximate numerical comparison and symbolic arithmetic. Although the conclusions of related experimental training studies [e.g., 23–26] on non-symbolic approximate numerical comparison and symbolic arithmetic have been questioned after several failed replication attempts with larger samples [21, 22, 27, 28], the methodological logic of such studies remains a promising tool with which to probe mechanistic relationships in numeracy development.

Here we adopt a similar experimental intervention strategy to investigate the role of symbols in early numeracy development. We compare the conceptual gains in numeracy of children who received non-symbolic numerical comparison training accompanied by different sorts of symbolic aids to those who received non-symbolic numerical comparison training alone (without symbolic aids). The training was delivered through a computer-based intervention, allowing us to manipulate the training content between children while tightly equating the training delivery and task demands. More broadly, our study develops and tests the feasibility of enhancing early numeracy through computer-based intervention, and the relative effectiveness of interventions providing different kinds of numerical content.

### Foundations for numeracy

Children have a rich repertoire of conceptual abilities that afford some forms of numerical representation well before entering school, including the ability to represent the approximate numerical magnitudes of sets and the ability to represent a limited number of individual objects in parallel [see 29–31 for reviews]. By the first year of life, infants detect numerical differences between sets of objects as long as the ratio differences are sufficiently large. For example, after watching, and losing interest in, a succession of images of 8 objects, 6-month-old infants regain interest when presented with an image of 16 objects, but their interest remains low when presented with a novel image of 8 or 12 objects [e.g., 32, 33]. By virtue of this approximate number system (ANS), young children and even infants have been shown to add and subtract numerical magnitudes [34]. For example, if 5 rectangles are covered by a screen and then 5 more rectangles move behind the screen, infants look longer if the removal of the screen reveals 5 rectangles (incorrect sum) rather than 10 [34]. However, as the name implies, these numerical representations and associated computations are imprecise, both for infants and for children who are presented with sets too numerous to count [35].

Children also have an object tracking system that allows them to represent and track a limited number of distinct objects in parallel, to distinguish one object from another (numerical identity), to compare sets for numerical equivalence and ordering using one-to-one correspondence, and to perform exact additions and subtractions of small numbers of objects [see 30 for a review]. For example, in a two alternative forced choice search procedure, 12-month-old infants have been shown to reliably approach a bucket with more food items compared to a bucket with fewer food items when the choice involves only a few, individually trackable items (e.g., 1 vs. 2, 2 vs. 3, or 1 vs. 3). However, they fail to reliably choose the bucket with more in cases of 1 vs. 4 or 2 vs. 4, suggesting an upper limit to the number of items that can be reliably tracked in parallel over occlusion [30, 36]. Infants also expect exactly three objects to stand behind a screen if the screen first was lowered over an array of two objects and then another object was placed behind the screen [37]. This object tracking system (OTS) appears to support children’s numerical comparison and addition by facilitating one-to-one matching of individual, trackable items. While it allows more precise comparisons, it is severely limited such that no more than three objects can be tracked at once.

The ANS and the OTS both are present from infancy and are hypothesized to play important roles in numeracy development [e.g., 30]. However, neither system appears to contain the information necessary to represent natural number concepts (i.e., the positive integers), accurately enumerate numbers greater than about 4, or perform exact arithmetic [see 29]. One potential reason for these limitations is that each system requires attention, and the two systems compete for attentional resources: attending to the numerical magnitude of a set reduces processing of the attributes of its individual members, whereas attending to one or more individual members reduces processing of the set’s numerical magnitude [31, 38]. Thus, the systems cannot be combined directly to determine the exact numerical concepts needed for numeracy. It is possible, however, that representations from the ANS and the OTS are combined by the use of numerical language or symbols [29, 39].

In addition to these non-verbal representational systems, children are endowed with a capacity for symbolic thought: a capacity that is seen most prominently in children’s learning of a natural language [e.g., 40] but that also is manifest in their increasing use of spatial symbols to convey complex meanings [see 41]. For example, children begin to learn the meanings of words before the end of the first year [e.g., 42–45]. By 2 years of age, children distinguish singular from plural noun phrases [e.g., 46, 47]. By 3 years, they begin to recite the ordered list of number words used for counting [1–3] and to comprehend and produce number words in quantified noun phrases [48].

In parallel, infants also begin to understand that items such as pictures can be used symbolically. Between the first and second year of life, infants shift from grasping at pictures as if they were objects themselves to pointing at them, suggesting an emerging understanding that the picture is meant to represent the object [49]. By the time children learn number words, they are able to use realistic and simple iconic models or pictures as maps to find hidden items, suggesting a further shift in symbolic thinking [e.g., 49–52]. These developmental shifts are thought to reflect an emerging understanding that physical items can be both objects in their own right and symbolic representations of something that is not currently present to their senses [e.g., 50].

### The role of symbols in numeracy development

There is a longstanding and nuanced debate regarding the role of symbols in the development of early numeracy, with a particular focus on the development of natural number concepts [e.g., 4, 29, 53–60]. Although a full discussion of the intricacies of this literature is beyond the scope of this paper, existing theories can be distinguished by whether they posit symbols as necessary for the development of natural number concepts or not. Nativist accounts posit that natural number concepts are innate, and symbols (e.g., number words) only serve to foster processes of enumeration and communication [e.g., 1, 53, 54, 61–64]. In contrast, other theories propose that symbols are critical for the development of natural number concepts [e.g., 29, 55–56, 58, 65, 66]. Some of these theories further posit a privileged role for linguistic symbols in the development of natural number concepts [39, 65, 66].

### Training numeracy

Despite some consensus among theories that symbols are important to early numerical development, few studies that we are aware of have tried to directly test the causal role of symbols in the emergence of numeracy through focused experimental interventions. This is not to say that early numeracy training studies with symbolic numbers have not been done. A number of other groups have attempted to improve early numeracy though symbolic number intervention, with some high-profile attempts showing promise [e.g., 12–16, 67, 68, but see 69]. However, most attempts, like many curricular interventions, involve broad training of a rich combination of symbolic and non-symbolic skills. That is, training was multifaceted, often including combinations of non-symbolic number, number words, digits, and/or the mapping between these symbolic and non-symbolic number forms and other magnitudes (especially spatial magnitudes). Given the multifaceted nature of these numeracy intervention studies, it is unclear which aspects of the training produced the changes in children’s numerical abilities.

In one exception, researchers directly contrasted outcomes of symbolic (digit) and non-symbolic numerical training in a small group of preschoolers [70]. Over 10 sessions of training, preschoolers in the experimental groups played computer games where they either associated Arabic digits or dot collections with physical magnitudes. Compared to a non-numerical control group, both the symbolic-digit training group and the non-symbolic training group improved on non-symbolic numerical skills. However, only those in the symbolic training group showed improvement on symbolic number skills, including exact symbolic arithmetic. These results suggest that symbolic number understanding may facilitate exact arithmetic.

### Current approach

**Fig 1:**
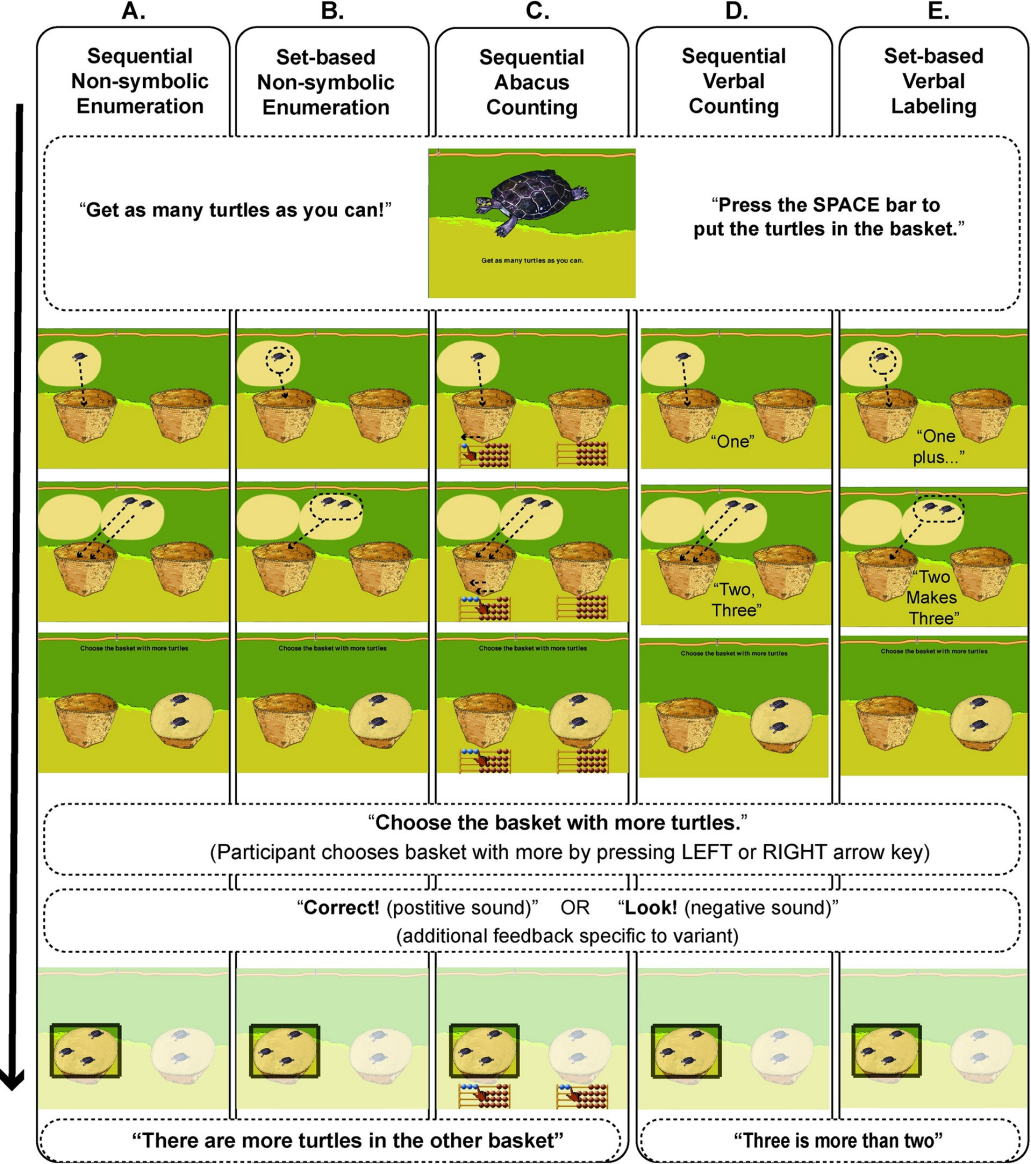
Schematic depiction of the basic training gameplay for the five training variants. Each column illustrates an example trial for each variant (A-E) where the child is asked to track a “1+2” event in the leftmost basket, and then to compare the sum (3) to the set of 2 items in the rightmost basket. Gameplay progresses from top to bottom (solid arrow). Dotted arrows indicate the animations of objects into the baskets, which could be sequential for each object (columns A, C and D) or simultaneous within each circled set of objects (columns B and E). Both the animation and the feedback period could be accompanied by verbal counting (column D) or numerical labeling (column E), or by a symbolic abacus where items moved in one-to-one correspondence with each added object (column C). Note the minimal differences between conditions and overlapping rows that indicate commonalities across the variants. Additional details of gameplay and feedback can be found in the text.

Here we report an initial, exploratory study using focused experimental training methods combined with a deep assessment of the outcomes to address the role of symbols in early numeracy. More specifically, children were assigned to play one of five variants of a numerical training game for about two weeks. All variants of the game involved tracking and comparing collections of objects on the basis of number and were equated for general gameplay, expectations about the training, and other cognitive and behavioral task demands. We chose non-symbolic numerical comparison as the basis of all training variants because a) it is numerical in nature b) it engages core systems available to all participants and c) non-symbolic numerical magnitudes and symbolic numbers become linked over development [e.g., 71–73]. Five different training variants presented different types of numerical content (see Fig 1A–1E, also see S1 Table in S1 File). Two of the training variants were designed to only emphasize non-symbolic, non-verbal representations of number [see 30, 31, 59]. In one, items were presented sequentially one-by-one to elicit attentional tracking of individual objects (OTS, Fig 1A). In the second, sets of items were presented simultaneously to elicit attention to approximate numerical magnitudes (ANS, Fig 1B). Two other training variants combined non-symbolic representations of number with linguistic number symbols. In one, items were presented sequentially and paired with verbal counting (Fig 1D). In the other, items were presented simultaneously as sets and paired with number word labels embedded within a noun phrase (Fig 1E). The latter variant was designed to associate non-symbolic representations of numerical magnitude with exact cardinal value labels by embedding this information in a richer linguistic context thought to be important for understanding of natural number. A final variant was designed to combine tracking of individual objects with a well-known system of spatial symbols for representing number and performing arithmetic: an abacus. In this condition, sequentially presented items were paired with an animated abacus that tallied items one-by-one (see Fig 1C).

Our methodology allowed substantial experimental control over the type of numerical input children received. By systematically combining groups of children trained on different variants and contrasting their outcomes [74], we tested several focused inferences about the role of symbols in numeracy development. Specifically, we contrasted the effects of training with or without symbolic aids, and training with or without verbal support (see S1 Table in S1 File). More broadly, the use of computerized and closely matched training allowed us to equate many aspects of the training procedure across participants, while minimizing the effects of particular teachers on the effectiveness of particular interventions [75].

In order to test the effects of these different types of training on numeracy, we systematically assessed several major components of early numeracy development before and after training rather than taking a single standardized summary outcome score. We assessed children’s symbolic number understanding through counting, their ability to perform non-symbolic numerical comparison, their sensitivity to one-to-one correspondence, and their ability to perform arithmetic transformations. These assessments served to identify the particular areas of numeracy, if any, that were affected by the different types of numerical training.

## Materials and methods

### Procedure overview

Children first were given pre-test assessments and those who had already mastered the critical numeracy skills to be trained were not invited to participate in the training portion of the study (see Participants section below for details). Children who had not yet mastered critical numeracy skills as assessed by the pre-test were pseudo-randomly assigned to play one of five variants of a computer-based numerical comparison training game over two weeks (see Fig 1). Assignment choices were made as the study progressed to keep groups approximately balanced in number of subjects who had completed testing, pre-test abilities, and age, based on pre-test and demographic data. Specifically, children were asked to play their assigned version of the computerized numerical training game twice a day for 9 days over a two-week period (total of 18 sessions). Each session was estimated to take about 15 minutes, although the game was self-paced. After two weeks of training, children were invited back for a post-test session with a majority of the same assessment tasks.

In order to obtain a large sample for this resource-intensive study, two investigators in different countries combined efforts to collect data on the effects of this numerical comparison training on early numeracy in preschool children. This approach yielded some variability in training environment (e.g., preschool vs. home training) and variability in demographics (country, age). All testing and training software and instruction were in English in the U.S. and Italian in Italy. The U.S. children were tested in the lab and in local preschools. The Italian children were tested in local preschools only. Those who were tested in the lab played their training game at home under the supervision of a parent or guardian. All children tested in preschools played their training game in a small group setting under the supervision of trained research assistants from the lab who provided one-on-one support to students as needed. In Italian preschools, all individuals within a single small group played the same training variant and variants differed between groups. In U.S. preschools, individuals within a single small group played different training variants. Regardless, all children tested in preschools in both countries wore headphones and were separated by at least a few feet to eliminate interference in variant-specific and individual player-specific feedback between laptops. All numeracy assessments were conducted one-on-one with children by trained research assistants, graduate students, or postdoctoral researchers. Although it was not always possible, our best efforts were made to have the same assessor conduct both pre- and post- testing sessions with a given child and remain naïve to the type of training the participant had received.

### Training

The training game presented all participants with the same basic gameplay scenario and objective: to track the number of items being placed in a basket, remember how many items were in that basket after occlusion, compare the number of items in that basket (occluded) with a second basket of items (visible), and to choose the basket with numerically more items. All five training variants started with a screen showing one exemplar item that served as a prompt to indicate the target items that the child would be working with during that session: fishes, turtles, birds, armadillos, bananas, or nuts. Target items varied from session to session. For trials with turtles as the target items, for example, the computer instructed participants to “Choose the basket with more turtles!”. The screen then updated to reveal two baskets to the left and right of the center of the screen and a set of turtles above the leftmost basket (see Fig 1). The number of items in the first set varied from trial to trial (see details on training variants below). Participants were instructed verbally by the computer to “Get as many turtles as you can. Press the SPACE bar to put the turtles in the basket (see Fig 1).” Participants then pressed the SPACE bar to activate an animation whereby the items moved into the leftmost basket and became occluded (Fig 1). Then a second set of target items appeared above the same leftmost basket and participants continued to press the SPACE bar to move the rest of the items from the second set in that same, leftmost basket (Fig 1). The number of items in this set could also vary from trial to trial (see details on training variants below). After all items from both sets where placed in the leftmost basket and occluded, an animation rotated the rightmost basket to reveal its contents (Fig 1). At that point, the participant, who could only see the contents of the rightmost basket, was instructed by the computer to “Choose the basket with more turtles!”. The participant then pressed the RIGHT or LEFT arrow key to choose the basket they thought had more turtles in it. Trial-by-trial feedback on accuracy was also provided; game difficulty increased across training sessions based on the participant’s individual intersession performance (Fig 1; see below the detailed description of feedback and progression for each variant). For all correct answers, a chime played, the computer said “correct”. For all incorrect answers, a trombone-like sound played, the computer said “look”. Additional feedback was provided depending on which training variant was being played (see details below).

#### Non-symbolic training variants

The first non-symbolic training variant, ***Sequential Non-symbolic Enumeration***, required participants to place items from the two subsets into the leftmost basket one-by-one by pressing the SPACE bar on the computer and to remember and compare the total number of items put into the leftmost basket to those revealed to be in the rightmost basket (Fig 1A). Training started with comparisons of 1–3 items in any one basket; the number of items to be compared increased both within and between sessions with the child’s performance, up to a maximum of 20 items per basket. Importantly, baskets always differed by only 1 or 2 items. The comparison difficulty progressed across sessions by increasing the total number of items to be compared. If the response was correct, a voice said “Correct!”. If the response was incorrect, the contents of both baskets became visible and a voice said: “Look! There are more (e.g.) turtles in the other basket.”.

The second non-symbolic training variant, ***Set-based Non-symbolic Enumeration***, required participants to place two sets of items in the leftmost basket by clicking the SPACE bar of the computer, but, in this case, items moved into the baskets as subsets (rather than one-by-one) (see Fig 1B). For example, if the first set of three items appeared at the beginning of the trial above the leftmost basket, pressing the SPACE bar moved the entire set of three items together into the leftmost basket. If the second set contained two more items, pressing the SPACE bar moved this second set of two items together into the same basket, making five total items that had been moved into and been occluded by the leftmost basket. Like the other variants, the rightmost basket then rotated to reveal its contents and the participant was prompted to choose the basket with more items. In contrast to the *Sequential Non-symbolic Enumeration* variant, all possible combinations of subsets adding up to the number of elements presented in the first basket were equally probable, and picked randomly from trial to trial (e.g., if, for one trial, the number of elements falling in the first basked was = 15, that could be implemented as 5 items in the first subset and 10 in the second subset, or 3 in the first subset and 12 in the second subset, etc.). The progression in difficulty for this variant was achieved by presenting numerical comparisons between baskets at ratios that were progressively closer to one rather than using progressively larger numbers of items like the *Set-based Non-symbolic Enumeration* variant. That is, the larger number/smaller number comparison ratios started off larger and therefore easier (larger basket: smaller basket average ratio = 1.6, e.g., as in comparing 8 to 14) and became smaller and therefore more difficult with the child’s performance across sessions (up to a max ratio of 1.12; e.g., as in comparing 8 to 9). The same corrective feedback of the other non-symbolic variant was given (“Correct!” for correct or “Look! There are more turtles in the other basket.” for incorrect response).

#### Symbolic training variants

There were three symbolic training variants, and they all also had the same basic gameplay scenario and numerical comparisons as the non-symbolic variants but included additional forms of symbolic aids to the numerical comparison. The actual numerical comparisons, set sizes, and resulting difficulty progression for all the symbolic variants matched the *Sequential Non-Symbolic Enumeration* variant.

Two of these symbolic variants employed verbal symbolic number aids. One verbal-symbolic variant, ***Sequential Verbal Counting*** was identical to the *Sequential Non-Symbolic Enumeration* variant except that items were verbally counted (i.e., one, two, three…) by the computer as they were placed by the participant one-by-one in the leftmost basket with the SPACE bar (during Fig 1D). The corrective feedback was also different in that the computer replayed the counting of the items of the first basket and then counted the items in the second basked, followed by a statement of the numerical relationship between cardinal values in each basket (e.g., “Three is more than two!”, see Fig 1D). In the feedback period, because items in the rightmost basket appeared together in the basket with no inherent cues for object individuation, every item was visually highlighted as the computer verbally counted them.

The other verbal symbolic variant, ***Set-based Verbal Labeling***, was identical to the *Sequential Verbal Counting* variant except that the items of the two subsets moved together as subsets (rather than item-by-item) into the leftmost basket and each subset was labeled with a cardinal value and embedded within a richer language context with the syntactic structure, “A plus B makes C” (with A and B referring to the subset cardinalities and C referring to their sum) (see Fig 1E). For example, if a first set of three items appeared at the beginning of the trial above the leftmost basket, pressing the SPACE bar moved the entire set of two items together into the leftmost basket and the computer would say “TWO”. If the second set contained one more item, pressing the SPACE bar would move this second set into the same leftmost basket together, and the computer would say “PLUS ONE MAKES THREE”. After this and like the other variants, the contents of the rightmost basket were revealed, and children were asked to choose the basket with more items. Corrective feedback involved repetition of the set-based verbal labeling and then a statement of the correct answer regarding the numerical relationship between the two baskets (e.g., in the context of the example above, if the rightmost basket contained two items and the child erroneously chose that basket as the most numerous, the feedback would be “TWO plus ONE makes THREE. THREE is more than TWO!”, see Fig 1E).

A final variant, the ***Sequential Abacus Counting (or Abacus)*** variant, was also identical in gameplay and numerical comparisons to *Sequential Verbal Counting* variant except that instead of verbal counting, a rudimentary visuo-spatial abacus was presented at the bottom of the computer screen that tallied items as they were placed one-by-one in the first basket (see Fig 1C). More specifically, as items were placed sequentially into the leftmost basket one-by-one by the participant using the SPACE bar, beads on the virtual abacus moved over by an animated hand from right to left, ending with the same number of total beads moved over to the left that had been placed in the leftmost basket (see Fig 1C). Participants were also provided a simple physical wooden abacus similar to the digital one and instructed to tally items in the rightmost basket after its contents were revealed). The participant then compared their tally on the physical abacus with the electronic tally of the first basket to choose the basket with more items. Importantly, this condition did not contain any number words. Corrective feedback involved a replay of the abacus tallying the first collection and then the second collection, after which the computer declared that there were more items in the other basket (i.e., the one they did not choose).

### Numeracy assessments

**Fig 2:**
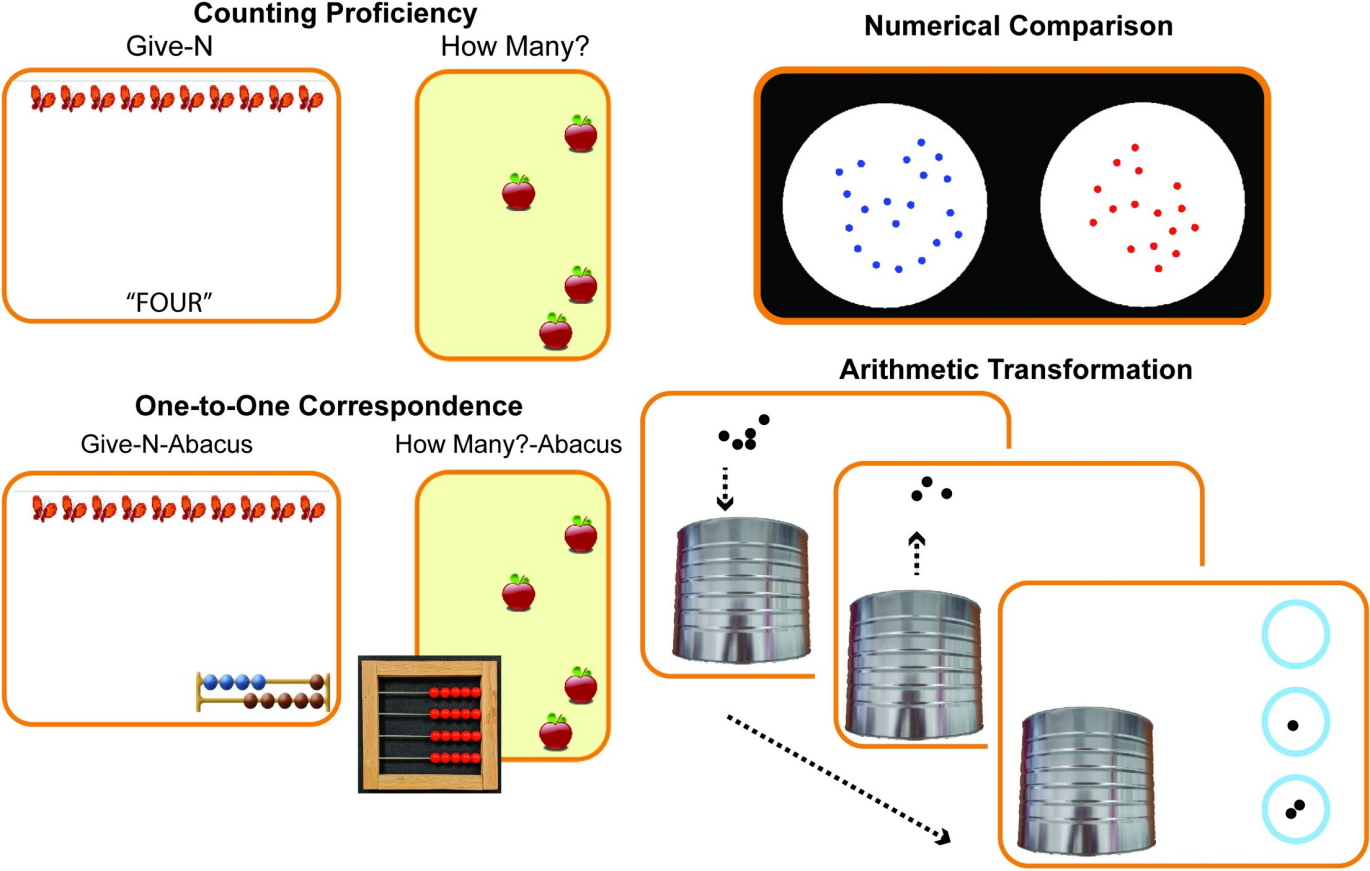
Depictions of numeracy assessment tasks. Counting proficiency was assessed using electronic versions of two well-known tasks designed to measure children’s ability precisely produce (Give-N) or enumerate (How Many?) sets of 1–8 items. On a single trial of the Give-N production task, children were asked to bring down a certain number of items (1–8) from a larger collection of 10 items at the top of the screen by pressing the SPACE bar. For the How Many? enumeration task, children were asked to report how many (1–8) items were presented on the screen. Numerical comparison was assessed by showing children a display containing two sets of dots side-by-side and asking them to point to the set with more dots. One-to-one correspondence was assessed by modified non-symbolic and non-verbal versions of our Give-N production and How Many? enumeration tasks, where verbal numbers were replaced by a non-verbal abacus. In the Give-N Abacus task, a visual representation of an abacus with a certain number of highlighted beads was presented at the bottom of the screen as the numerical prompt instead of a number word. Children were asked to match the same number of items as were on the abacus by bringing them to the bottom of the screen through pressing the SPACE bar. For the How many? Abacus task, children were presented with pictures containing 1 to 8 items on the computer screen and were asked to match the number of items on the computer screen by moving over the same number of beads on a physical abacus they were given. Arithmetic transformation was assessed using a non-symbolic subtraction task where 1–6 items entered a can via animation (e.g., 5), some or all of those items came out (e.g., 3), and then children were asked to choose how many items they thought were left in the can by pointing to the options of “zero”, “one”, or “two” items on the right part of the screen. Additional parameters of all tasks can be found in the main text. PLEASE NOTE: Figs are not to visual scale with actual assessment tasks.

Four main aspects of early numeracy were assessed pre- and post-training: counting proficiency, approximate numerical comparison, one-to-one correspondence, and exact arithmetic with objects (see Fig 2). The tasks were administered in electronic form on a laptop computer in a fixed order for the majority of participants. Computerized versions were used in order to provide more experimental control over the testing, and, as a result make testing more comparable across children, experimenters, and testing sites. Deviations in order, although rare, were required occasionally due to the logistics of testing individual children. These particular assessment tasks have been previously described in several recent publications focusing on relationships between pre-test variables only [72, 75–77]; no published report to date has analyzed post-test data or the effects of training with this dataset.

#### Counting proficiency

The understanding of how to use counting to produce or enumerate the exact cardinality of sets was assessed using electronic versions of two well-known tasks designed to measure children’s ability to precisely enumerate or produce sets of 1–8 items: Give-a-number (or Give-N) [e.g., 3]. and How Many? tasks [e.g., 78]. For the Give-a-number task children were presented with 10 items (e.g., butterflies) at the top of a computer screen and asked to give a certain number of them to the experimenter by bringing them to the bottom of the screen by pressing the SPACE bar (see Fig 2). Each press of the space bar brought one of the items from the top to the middle of the screen in an animated motion. The child was told to press the ENTER button once the correct number of items had been “given” in this fashion. If the child indicated that she had made a mistake, the animals were reset the top and the trial was started over. The task always started with a request for “one” and this trial was completed with the experimenter as a practice trial with feedback to ensure the child understood the task. After this, 7 more trials presented requesting each number “two” through “eight” in a random order. A total proportion correct out of 8 was taken as the accuracy score for this task (with trial 1 given as correct for all children).

For the How many? task, children were presented with pictures containing 1 to 8 items (e.g., apples) on the computer screen. Children were asked to enumerate the items. For example, in a trial showing apples, children were asked “How many apples? Can you count them?” (see Fig 2). The last number word children produced in the counting process was entered into the computer by the experimenter by pressing the corresponding number key on the keyboard. The task started with 1 item, and children received corrective feedback from the experimenter for this trial (e.g., “yes/no, there is one apple”). After this practice trial, sets between 1 and 8 items were presented in a random order. A total proportion correct out of 9 (including the first trial with 1) was taken as the accuracy score for this task.

It should be noted that a subset of children completed two more rounds of each of the two counting proficiency tasks. However, only performance on the first round was analyzed to make scores comparable across the entire dataset. Counting proficiency was calculated as the average proportion correct across the first run of each of the two tasks. First round accuracy data from each task was highly correlated with accuracy data when calculated from three rounds for each number per task in the subset of children with additional rounds (r(102) = .927, p < .001).

From performance on these tasks, we also derived a continuous estimate of knower-level (KL), or the highest number known between 0–8. Knower-level is often treated as a categorical variable and often derived from a titration method for each child [see 29]. However, given the nature of our data (all children tested on numbers 1–8) and a priority for analyzing data continuously in a similar fashion to our other metrics, we took a slightly different approach to calculating knower-level. Specifically, we identified the highest consecutive number that the child answered correctly from 1–8 with the possible exception of one, non-sequential error (i.e., an error followed by a correct response on the next number). This was done independently for the Give-a-Number task and the How Many? task and then scores on each task were averaged to derive a single KL for each child. Knower-levels calculated from a single round of data in each task were highly correlated with knower-levels when calculated from 3 trials for each number per task in the subset of children with additional rounds (r(101) = .833, p < .001).

#### Approximate numerical comparison

Understanding of the relative magnitude relationship between sets was assessed using a non-symbolic, approximate number comparison task [e.g., 72, 79]. In this task, children were shown a display containing two sets of dots side-by-side, one with blue dots and the other with red dots. Children were asked to point to the set with more dots (see Fig 2). The number of dots in a given set ranged from 8 to 32, with one set always containing 16 dots. Seven ratios of comparison (1:2, 2:3, 3:4, 4:5, 13:16, 7:8, and 8:9) were used to create different levels of comparison difficulty. The factors of side of the blue colored dots and the side with the larger set were counterbalanced across the trials and ratio of comparison was randomized across the task. Although randomly presented, over the course of the task half of trials contained sets equated on individual dot size, where the number of dots was positively correlated with total area of the dots in the array, or congruent with non-numerical spatial properties (i.e., congruent trials). The other half of trials contained sets equated on total area, where the number of dots was inversely related to size of the dots, or incongruent with nonnumerical spatial properties (i.e., incongruent trials) [see 80 for logic of this control]. This design served to discourage children’s use of non-numerical features of the sets in making their judgments. To help children understand and engage with the task, the first 10 trials included corrective feedback in the form of a cartoon smiling face or frowning face for correct and incorrect answers. The remaining 54 trials had no feedback. A total percent correct (out of 64 trials) was used as the main score for the test.

#### One-to-one correspondence

The ability to precisely match sets of items based on number was accessed using modified non-symbolic and non-verbal versions of our counting proficiency tasks: The Give-a-number Abacus task and the How Many? Abacus task. For the Give-a-number Abacus task children were presented with 10 items (e.g., butterflies) at the top of a computer screen. On each trial, a visual representation of a rudimentary abacus with a certain number of highlighted beads was presented at the bottom of the screen as the numerical prompt (see Fig 2). Children were asked to give the experimenter the same number of items (e.g., butterflies) as were on the abacus by bringing them to the bottom of the screen through pressing the SPACE bar. Each press of the space bar brought one of the items from the top to the bottom of the screen. Children were instructed to press the ENTER button when they had matched the number of items to the number of highlighted beads on the abacus. The task always started with an abacus with 1 highlighted bead and this trial was completed with the experimenter as a practice trial with feedback to ensure the child understood the task. After this, 7 more trials presented requesting each 2–8 items in a random order. A total proportion correct out of 8 was taken as the accuracy score for this task. (with the first practice trial counted as correct for all children).

For the How many? Abacus task, children were presented with pictures containing 1 to 8 items (e.g., apples) on the computer screen and were asked to match the number of items on the computers screen by moving over the same number of beads on a physical abacus they were given (see picture of abacus in Fig 2). The task started with 1 item, and children received corrective feedback from the experimenter for this trial (e.g., “yes, there are the same number of apples and beads/no, there are not the same number of apples and beads. Let’s fix it.). After this practice trial, sets between 1 and 8 items were presented in a random order. A total proportion correct out of 9 (including the first trial with 1) was taken as the accuracy score for this task.

Only exact matching between sets based on number (e.g., get the same number; make them the same) was encouraged by experimenters in the instruction and practice trial. Counting or specific number words were not mentioned (although they were not disallowed as a strategy if used spontaneously), and children were allowed to use whatever strategies they wanted to provide answers. It should be noted that a subset of children completed two more rounds of each of the two abacus tasks. However, only performance on the first round was analyzed to make scores comparable across the entire dataset. One-to-one correspondence ability was calculated as the average proportion correct across the first run of each of the two tasks. One-to-one correspondence accuracy calculated from a single round of data in each task was highly correlated with one-to-one correspondence accuracy data calculated from three rounds of data in the subset of children with additional rounds (r(94) = .924, p < .001).

#### Arithmetic transformation

The ability to compute the effects of an arithmetic transformation on the exact cardinal value of a set of items was assessed using an exact non-symbolic subtraction task similar to that used in other studies of non-symbolic arithmetic [e.g., 81, 82]. In this task, children were shown a collection of 1–6 items (i.e., black dots) at the top of the screen (see Fig 2). The child was encouraged to focus on how many items were at the top of the screen (e.g., “see” or “look”). When the child was ready, the experimenter pressed the SPACE bar and said “some go in”, pressed it again and said “some come out”, and then asked how many are left in the bucket? To aid the child, images containing 0, 1, and 2 dots appeared on the right side of the screen. Children were asked to point the number of dots left in the bucket (e.g., “zero”, “one”, or “two”). Children received 15 trials of the exact subtraction task. Six trials started with smaller numbers 1–3 and nine trials started with larger numbers 4–6. The order of trial presentation was random. Exact non-symbolic arithmetic ability was defined as the proportion correct out of 15 trials.

### Other variables of interest

#### Age

Either through parental report or the preschool we obtained the child’s age at time of pre-testing.

#### Knowledge of the count list

Count list knowledge was measured as a proxy of number-specific experience [see 72, 83]. To assess this, children were asked to recite the count list as high as they could starting with “one”. In order to conserve time, children were stopped if they successfully surpassed the number “twenty-five”. Their score was the highest consecutively correct number recited with a minimum of 1 and maximum of 25.

#### Vocabulary

Children’s receptive vocabulary was assessed using a modified version of the Peabody Picture Vocabulary Task [84]. In this task, children were presented with a target word spoken by the experimenter and were asked to identify the corresponding picture among a field of 4 potential choices. Children in the U.S. received the standardized protocol for test administration [see 72, 84 for details). Due to time constraints, a shortened version was administered in testing the Italian sample. In this case, children were only given 4 vocabulary lists corresponding to the starting point for ages 5–8 (PPVT lists 4–7). In order to make the data from this task comparable across samples, the same reduced raw score was calculated for all participants. That is, children were assigned a score of 1–5 based on the first list (out of PPVT lists 4–7) where they made 2 or more errors. A score of 5 was only given if they made less than 2 errors on all lists (1–4) including the last and most difficult list. Decisions on which sets to use and how to create a reduced score were based on pre-study piloting. As verification that this reduced score was meaningful, the reduced score and full-scale raw scores in the U.S. sample were strongly correlated (r(133) = .659, p < .001).

#### Unused demographic variables

Some additional demographic variables (e.g., years in preschool, parent education, alphabet knowledge, parent income) were collected in different subsets of the data. However, these additional demographic variables were systematically missing in at least 20% of the dataset (often much more) and due mostly to differences in data collection protocols between subsamples (e.g., parent education in U.S. lab-based sample, but not in preschools in the U.S. or Italy). Given that we wished to combine subsamples, these additional demographic variables were systematically collected in some subsamples and not others, and different data collection protocols in different subsamples resulted in a large portion of systematically missing data, we did not include them in our analyses.

### Participants

A total of 215 children participated in pre-testing, training, and post-testing and had complete data for the first numeracy assessment (i.e., counting proficiency). Fewer children had complete data for the other assessments (numerical comparison n = 214, one-to-one correspondence n = 170, arithmetic n = 177, see S2 Table in S1 File for more details) due to either an individual child not completing that particular assessment during both testing sessions or a particular task not being run at a particular testing site. Children in the final dataset were, on average, 49.15 months of age (SD = 7.82 months, range = 35.97–76.01 months). Data was collected in the U.S. and Italy. Children in Italy were on average 8.53 months older (n = 79, M = 54.54 months) than preschool children tested in the U.S (n = 136, M = 46.01 months). The range of participant ages was also greater in Italy (range = 40 months; 36–76 months) than the U.S. (range = 21.47 months; 35.97–57.44 months). Children in the U.S. sample also reported less years in preschool (M = .56 years, SD = .61 years) than children in Italy (M = 2.10 years, SD = .84 years), although these estimates are likely biased due to a substantial amount of missing (48/215 missing). Country differences in age, and possibly differences in years in preschool, are not an artifact, but due to the fact that children in this part of Italy tend to start elementary school later than children in U.S.

#### Data inclusion

In order to be included in the analysis, children had to participate in pre-, training, and post-testing (S1 Fig in S1 File). Children who were more than 90% accurate in counting proficiency (see Methods and Materials below for operational definition) at pre-test were considered too advanced for the training as pre-specified in the original grant proposal. In the lab, our best efforts were made to not enroll these highly accurate children in training at all. For a variety of reasons, however, some children in the lab were enrolled in training despite pre-test mastery (e.g., experimenter mistake, parent/child really wanted to do the training, twin sibling did qualify for the training). For preschool-based testing, participation policies at some testing locations required inclusion of all children despite ceiling-level performance. Regardless of the reason, children who were more than 90% accuracy in the counting proficiency assessment were eliminated from analysis, as they were considered to be too advanced for the training with less prospect for improvement.

An additional 197 children participated in the pre-test portion of the study but did not finish it (n = 19), were miscategorized as too good to participate and therefore not invited to participate in training (n = 4), decided not to participate in the training portion of the study after the pre-test (n = 8), or were too good to be included in the analysis (greater than or equal to 90% accuracy on counting proficiency, n = 166, S1 Fig in S1 File). Of those not included because of ceiling-level performance, 100 did not participate in training or post-testing at all and 66 participated in the training and post-testing but were excluded at the data cleaning stage (S1 Fig in S1 File). Again, as mentioned above, many of these children participated in the training due to their preschool participation policies even though they were too good for inclusion in the final analysis.

An additional 28 children enrolled in the training, but never completed any training or post-test (n = 5), did some training but did not return to the lab for a post-test or were absent from preschool on the day of the post-test (n = 20), or completed training and were present for the post-test but did not complete minimum required post-test assessments to be included (n = 3).

An additional 33 children were assigned to participate in a non-numerical control training condition, but it was found out after the fact that many of these children were not assigned to the control condition using the same procedure that was used in assignment to the experimental conditions. As a result, the integrity of this control condition as a comparison to the experimental training groups was compromised, and it was not included in the analyses.

Finally, 9 additional participants were excluded from the final dataset for the following specific reasons: parental report of developmental or neurological issue (n = 4), not being native speakers of the training game language (English or Italian, n = 1), missing data critical to categorizing training condition (n = 1), incorrect birthdate information/already being enrolled in elementary school (n = 1), actively disregarding the training instructions (n = 1), actively participating simultaneously in lab and preschool versions of the study (n = 1).

#### Recruitment and compensation

All children were recruited through local community events, word of mouth, or through their own preschool. Written informed consent to participate was obtained from a parent or guardian of child participants either in-person in the lab or through sending consent forms home with children from preschool and asking that they return them signed if they agreed to allow their child to participate. The study was approved and supervised by the University of Illinois Office for the Protection of Research Subjects and the University of Trento Ethical Committee. For lab studies, children were given a small appreciation gift (e.g., book, t-shirt, toy, certificate of participation) and parents were given travel reimbursement of 15 dollars or a gift card for the same amount for each testing session.

### Analyses

First, we explored the summary characteristics of our data by analyzing the relationships between demographic factors, pre-test scores, and gains after training (post-test scores minus pre-test scores) using basic descriptive statistics, simple correlations, and partial correlations. Second, we analyzed the extent to training in general, regardless of training variant, led to gains in numeracy using t-tests and the extent to which these gains were related to the demographic factors and performance on the training itself using partial correlations. Third, we tested several focused hypotheses regarding the hypothesized mechanisms that may drive numeracy development. Given the resources required to run this study, including the intensity of training and comprehensive scope of pre- and post- training assessment, it was not possible to obtain large enough samples to yield high-powered pairwise statistical comparisons between all five individual training variations as we had originally hoped. However, different variants were able to be conceptually grouped in different ways to explore several hypotheses regarding the role of symbols in numeracy development, as indicated in the introduction [see 74 for similar combinatorial approach]. Sensitivity analyses conducted afterwards suggested that our approach should be able to detect medium effect sizes with a high degree of power (.95) given our statistical design and our obtained sample sizes (see S2 Fig; S2 Text in S1 File).

The first focused hypothesis we tested was that training combining numerical comparisons with symbolic aids of any sort (verbal or visuo-spatial) would lead to more improvement than training with non-symbolic numerical comparisons alone (i.e., without symbolic aids). To test this hypothesis, we compared the outcomes of the variants trained with symbolic aids (verbal-counting; verbal-labeling; abacus-counting, n range = 92–130 depending on assessment task) to those trained with non-symbolic numerical comparison only (sequential non-symbolic and set-based non-symbolic enumeration, n range = 70–85 depending on assessment task).

The second focused hypothesis we tested was that numerical comparisons with verbal number aids specifically lead to more improvement than numerical comparisons without verbal number aids. We tested this hypothesis in several ways. First, we contrasted the outcomes of children who were trained on variants involving verbal numbers (verbal-counting; verbal-labeling, n range = 58–93) with children trained on all the other variants (n range = 103–122). Second, we restricted our analyses to compare outcomes in only those children that received a symbolic variant, contrasting those trained with verbal-symbolic numbers (verbal counting and verbal labeling, n range = 58–93) to those who received non-verbal symbolic abacus training (abacus counting, n range = 34–37). Given the small sample of those trained on the abacus counting variant, we interpret any pairwise comparisons with this single condition with more caution. Finally, we conducted several additional exploratory analyses to better understand the symbolic training advantages that we observed. We also visualize gains for individual training variations and include those visualizations in the supporting information (S3 Fig in S1 File).

We tested for training effects using ANCOVAs with the planned combinations of training variants entered as categorical independent variables and the post-test scores as the dependent measure. Pre-test scores were always entered as a covariate in ANCOVA models to account for any differences in starting point before training, allowing us to make inferences about gains due to training. In cases of significant effects, we determined whether there were any additional pre-training demographic differences between the particular groupings being contrasted that could alternatively explain the difference (see S1 Text in S1 File). Factors on which groupings differed, if any, were then were added to determine if significant results held after controlling for those differences as covariates (i.e., in age, vocabulary, and/or experience with symbolic numbers as tested through count list knowledge). We used pre-post difference scores (post-test minus pre-test scores) for figures in order to more intuitively interpret and compare gains between training groupings and tasks.

## Results

### Pre-test

Children started with a wide range of numerical abilities before training (counting proficiency M = 59.45%, SD = 20.93%, range 6%-88%; numerical comparison M = 63.55%, SD = 11.08%, range = 39%-94%; arithmetic M = 39.16%, SD = 17.15%, range = 8%-92%; one-to-one correspondence M = 54.63%, SD = 21.20%, range = 13%-100%). Correlations between demographic variables and pre-training abilities revealed that all numeracy measures were related to age, count list knowledge, and vocabulary before training (i.e., pre-test; Table 1).

**Table 1:** Pre-training correlations between variables. Pearson’s correlation coefficient (r), p value, and number of participants included in the analysis reported in each cell.

Variables	Age	Count List	Vocabulary	Count. Prof.	Comparison	Arithmetic	One-to-one
Age	---						
	---						
	---						
Count List	.336	---					
	< .001	---					
	211	---					
Vocabulary	.375	.322	---				
	< .001	< .001	---				
	210	210	---				
Count. Prof.	.299	.504	.274	---			
	< .001	< .001	< .001	---			
	215	211	210	---			
Comparison	.336	.480	.346	.429	---		
	< .001	< .001	< .001	< .001	---		
	214	210	209	214	---		
Arithmetic	.270	.213	.150	.251	.213	---	
	< .001	.005	.049	.001	.005	---	
	177	173	172	177	176	---	
One-to-One	.589	.392	.389	.514	.380	.370	---
	< .001	< .001	< .001	< .001	< .001	< .001	---
	170	167	167	170	169	167	---

There were some differences in pre-test scores between samples, with Italian children showing better pre-test performance in arithmetic (F(1,175) = 5.125, p = .025, partial eta squared = .028), and one-to-one correspondence (F(1,168) = 40.114, p < .001, partial eta squared = .193) compared to children in the U.S. sample. There were no differences between samples in counting proficiency (F(1,213) = .320, p = .572, partial eta squared = .002), numerical comparison (F(1,212) = .072, p = .789, partial eta squared < .001), or knower-level (F (1,210) = .231, p = .631, partial eta squared = .001) at the pre-test.

### Training

Children were compliant with the training instructions, completing an average of 211.67 out of 280 planned trials (SD = 67.82, Range = 24–522) and an average of 14.49 (SD = 4.36, Range = 2–25) out of 18 planned sessions of the training game over the course of the two weeks. On average, they also performed fairly well, answering correctly 71.96% (SD = 11.08%, Range = 48%-99%) of the problems they attempted. Finally, children made reasonable progress through the training game, reaching an average level of 10.11 (SD = 2.74, Range = 0–15) out of a possible 15 levels.

### Overall outcomes

On average, children showed improvement in all the numerical outcome measures after training except approximate numerical comparison (t(210) = 1.209, p = .228, M gain = 0.82%, SD = 9.81%). That is, children’s gains were significantly different from zero in counting proficiency (M gain = 11.60%, SD = 17.50%, t(214) = 9.716, p < .001), one-to-one correspondence (M gain = 5.98%, SD = 16.38%, t(160) = 4.619, p < .001), arithmetic (M gain = 4.98%, SD = 17.81%, t(172) = 3.679, p < .001), and knower-level (M gain = 1.05 knower levels, SD = 1.75, t(203) = 8.593, p < .001).

An analysis of the relationship between performance on the actual training and post-training numeracy outcomes controlling for pre-test scores using partial correlations revealed a number of effects. Training compliance (total number of trials completed) was related to improvements in counting proficiency (r(212) = .141, p = .039) and corresponding knower-level (r(201) = .192, p = .006). Proportion of correct answers on training was related to post-training improvements on all assessment tasks (numerical comparison r(208) = .325, p < .001; counting proficiency r(211) = .229, p = .001; one-to-one correspondence r(158) = .264, p = .001; arithmetic r(170) = .192, p = .012; knower-level r(201) = .181, p = .010). Highest level of difficulty reached during training was also related to post-training improvements on numerical comparison (r(200) = .197, p = .005) and to counting proficiency (r(203) = .144, p = .040). Together these results link actual performance during training to various improvements in numeracy after training.

An analysis of the relationship between demographic factors (age, vocabulary, count list knowledge) and post-training outcomes accounting for pre-test scores through partial correlations also revealed a number of effects. Age was positively related to gains on all assessments of numeracy except numerical comparison (numerical comparison, r(208) = -.023, p = .735; counting proficiency, r(212) = .280, p < .001; one-to-one correspondence, r(158) = .195, p = .014; arithmetic, r(170) = .185, p = .015; knower-level, r(201) = .225, p = .001). This finding indicates that our training was overall more effective for older compared to younger children within our sample. Vocabulary was also related to gains in counting proficiency (r(207) = .171, p = .013) and corresponding knower-level (r(198) = .199, p = .005). This indicates that children demonstrating better receptive vocabulary at pre-test improved more in counting through training. Knowledge of the count list at pre-test was positively correlated with post-training gains in counting proficiency (r(208) = .280, p < .001), corresponding knower-level (r(199) = .304, p < .001), and one-to-one correspondence (r(158) = .304, p < .001). This finding indicates that the more familiar children were with the count list at pre-test, the greater amount of gain they made in counting proficiency and one-to-one correspondence after training.

The further breakdown by sample showed that Italian children performed better than US children in counting proficiency and arithmetic after training, even accounting for pre-test scores as a covariate (counting proficiency: F(1,212) = 10.272, p = .002, partial eta squared = .046; arithmetic: F(1,170) = 4.265, p = .040, partial eta squared = .024). However, these effects were eliminated when further accounting for age as a covariate (counting proficiency: F(1,211) = 1.079, p = .300, partial eta squared = .005; arithmetic: F(1,169) = 0.927, p = .337, partial eta squared = .005), with age being a significant covariate in the counting proficiency model for counting proficiency (F(1,211) = 8.545, p = .004, partial eta squared = .039). These analyses suggest that differences in the effects of training between the Italian and U.S. children can likely be explained by age differences rather than geographic or cultural differences between the sub-samples. Gains did not differ across the Italian and the US samples for the other aspects of numeracy assessed (numerical comparison: F(1,208) = 1.126, p = .290, partial eta squared = .005; one-to-one: F(1,158) = 1.085, p = .299, partial eta squared = .007; knower-level: F(1,201) = 3.725, p = .055, partial eta squared = .018).

### Focused analyses of mechanism

The main purpose of our study was to explore several focused hypotheses regarding the mechanisms that drive early numeracy and their particular effects by contrasting different variants of the training.

#### Symbolic versus non-symbolic training

**Fig 3:**
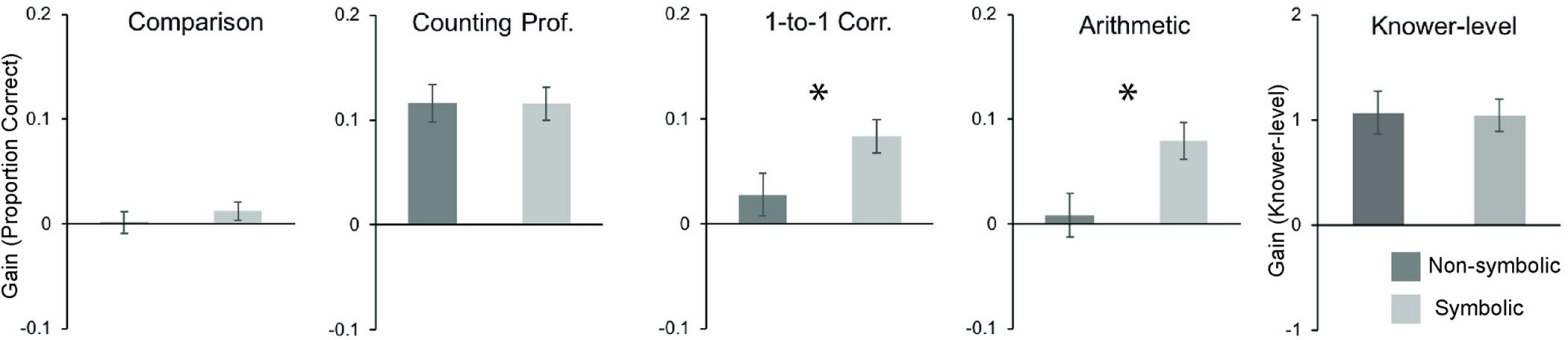
Mean gains in numeracy after numerical training with or without symbolic aids. Gains calculated as post-test score minus pre-test score. Error bars are -/+ 1 SE. * indicates statistically significant differences between numerical comparison training with and without symbolic number aids.

In our first focused analysis we asked whether numerical comparison training with symbolic aids (abacus counting, verbal counting, or verbal labeling, n = 69–85) was more effective than training with non-symbolic numerical comparison alone (set-based or sequential non-symbolic enumeration, n = 92–130). The analyses showed that children trained on numerical comparisons with symbolic aids made larger gains on two of the four outcome measures, one-to-one correspondence (F(1,158) = 7.851, p = .006, partial eta squared = .047) and arithmetic (F(1,170) = 6.260, p = .013, partial eta squared = .036), compared to children who participated in non-symbolic numerical comparison training (Fig 3). These outcomes could not be explained by pre-test differences in the groups or performance differences on the training task itself (see S1 Text in S1 File). There were no differences in improvement between the two training groups on numerical comparison (F(1,208) = 1.982, p = .161, partial eta squared = .009) or counting proficiency (F(1,212) = 0.003, p = .958, partial eta squared < .001). There were also no differences when counting proficiency was coded as a knower-level. (F(1,201) = 0.003, p = .955, partial eta squared < .001). These results show that numerical comparison training with symbolic aids leads to greater increases in several important aspects of numeracy compared to non-symbolic numerical comparison training alone.

#### Verbal versus non-verbal training

A second focused question concerned whether training variants involving verbal numerical aids conferred a unique advantage over non-verbal training variants. Several analyses were conducted to investigate this possibility. First, we contrasted outcomes of those whose training involved verbal numbers (verbal counting & verbal labeling, n = 58–93) to all others (non-symbolic sequential, non-symbolic sets, & abacus counting, n = 103–122). These comparisons showed that children whose training highlighted verbal numbers did not show greater improvements than those who were trained with nonverbal variants. That is, there were no differences in accuracy at post-test, accounting for pre-test scores, on any of the numeracy assessments between verbal and non-verbal training types (counting proficiency: F(1,212) = 1.545 p = .215, partial eta squared = .007; numerical comparison: F(1,208) = 0.250, p = .617, partial eta squared = .001; arithmetic: F(1,170) = 3.120 p = .079, partial eta squared = .018; one-to-one correspondence: F(1,158) = 0.711, p = .400, partial eta squared = .004). There were also no differences between verbal and nonverbal training when counting proficiency was coded as knower-levels (F(1,201) = 1.850, p = .175, partial eta squared = .009).

**Fig 4:**
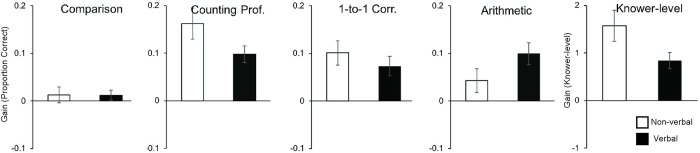
Mean gains in numeracy after numerical comparison training with symbolic verbal or symbolic non-verbal aids. Error bars are -/+ 1 SE. Note that gains themselves were significantly different from zero in all areas except numerical comparison, but the differences between verbal and non-verbal symbols on numerical comparison, counting proficiency, one-to-one correspondence, and arithmetic were not significant. A moderate advantage for the non-verbal symbolic aid (abacus) on knower-level.

Next, we compared outcomes of those who participated in the training with symbolic verbal number aids (verbal counting & verbal labeling, n = 58–93 depending on task) to those who participated in training with the symbolic but non-verbal abacus aid (abacus counting, n = 34–37). There were no clear differences in outcomes on any of the numeracy assessments between verbal and non-verbal symbolic training types either (counting proficiency: F(1,127) = 3.129 p = .079, partial eta squared = .024; numerical comparison: F(1,126) = 0.462, p = .498, partial eta squared = .004; arithmetic: F(1,98) = 0.076, p = .783, partial eta squared = .001; one-to-one correspondence: F(1,89) = 1.724, p = .193, partial eta squared = .019; see Fig 4). There was a small difference between verbal and non-verbal symbolic training when counting proficiency was coded as knower-levels (F(1,121) = 4.298, p = .040, partial eta squared = .034), with non-verbal symbolic abacus training leading to higher knower-levels at post-test than verbal-symbolic training after accounting for pre-test abilities. However, this effect was eliminated when age and/or vocabulary differences between groups (see S1 Text in S1 File) were accounted for as additional covariates (ps > .27).

Finally, to further explore whether the observed advantages of symbolic training were driven by the children that completed verbal number training or whether the benefits of symbolic training may extend to non-verbal symbolic visual-spatial aids like the abacus, we compared outcomes of those whose training involved verbal-symbolic aids and those in the non-verbal symbolic (abacus) training group separately to the outcomes of those trained on non-symbolic numerical comparison on the two measures where a symbolic training advantage had been observed: arithmetic and one-to-one correspondence. One-to-one correspondence accuracy improved more for those trained with the non-verbal symbolic abacus (n = 34) compared to those trained on non-symbolic numerical comparison alone (n = 69, F(1,100) = 9.035, p = .003, partial eta squared = .083), while there was no clear differences in one-to-one correspondence outcomes between those trained on numerical comparison with verbal-symbolic aids (n = 58) and those trained on non-symbolic numerical comparison alone (F(1,124) = 3.549, p = .062, partial eta squared = .028). There results could not be accounted for by pre-test differences (see S1 Text in S1 File) and results held when controlling for grouping differences in age as an additional covariate (p = .010; p = .063). The reverse seemed to be true for improvements on arithmetic. Improvement on arithmetic was marginally greater for those trained with verbal-symbolic aids (n = 65) compared to those trained on non-symbolic numerical comparison alone (n = 72, F(1,134) = 4.195, p = .042, partial eta squared = .030), but there was no difference in arithmetic outcomes between those who trained with the abacus (n = 36) and those who trained on non-symbolic numerical comparison alone (F(1,105) = 2.665, p = .106, partial eta squared = .025). These exploratory results suggest that gains on one-to-one correspondence may have been driven by those trained to tally with the abacus, whereas gains in arithmetic may have been driven by those whose training involved verbal numbers.

## Conclusions

There has been a surge of interest in improving the numerical abilities of children before entering school, as it is clear that preschool numeracy is a strong predictor of academic and professional success throughout life [e.g., 7, 8]. There is also a longstanding interest by psychologists in discovering the cognitive mechanisms that drive early numeracy [e.g., 1, 29, 56, 39, 85]. Although intuitively important, central to most cognitive current theories, and a part of most informal and formal preschool curricula, the precise role of symbol use in the emergence of numeracy is not well understood. Here we began to experimentally investigate the role of symbols in numeracy development through a controlled experimental training study.

### Feasibility of numerical comparison as an intervention tool

We first assessed the overall feasibility of using our particular instantiation of numerical comparison as an intervention tool for enhancing numeracy in preschool aged children. We found that children were compliant and successful on the training game itself. Furthermore, participating in the numerical comparison training was associated with significant gains in three of the four targeted areas of numeracy. That is, on average, children improved in counting proficiency, one-to-one correspondence, and exact arithmetic calculation with objects. Demographic variables of age, vocabulary, and pre-test count-list knowledge, as well as performance during actual training, were all positively related to improvements in numeracy, suggesting our training may have been more effective in older children with more advanced vocabularies and knowledge of the count list, but who had not yet mastered counting.

Although test-retest improvement or ongoing cognitive development may have accounted for some of the observed gains from sessions 1 to 2, several pieces of additional evidence suggest that the numerical training itself also contributed. First, participants made about one “knower-level” gain over the course of two weeks—such a gain normally takes at least several months on a typical developmental timescale [see 3]. Second, these gains were systematic rather than randomly distributed. That is, to make a gain in knower-level, one must improve sequentially in number knowledge performance while retaining performance on previously mastered numbers from pre-test to post-test. As such, systematic gains in knower-level are less likely to result for simple test-retest improvements, where gains would more likely be distributed randomly across trials. Third, individual differences in improvements in numeracy were related to individual differences in training performance, further suggesting that the training itself was driving gains.

### The role of symbols in numeracy development

Next, we tested the role of symbols in early numeracy, comparing performance of children that received numerical comparison training with symbolic aid(s) to children who received non-symbolic numerical comparison training alone. Here we provide novel experimental evidence of how symbolic aids may enhance numeracy.

Children who participated in training that combined their non-verbal numerical intuitions with symbolic numerical tools made significantly larger gains on the two non-verbal conceptual assessments of exact number understanding, compared to those children trained on non-symbolic numerical comparison alone. More specifically, children whose numerical comparison training involved using a symbolic abacus, verbal counting, or verbal-numerical set labeling made larger gains on tests of one-to-one correspondence and exact arithmetic with objects than children who were trained with a comparably demanding task that only involved comparing arrays of objects without any additional symbolic-numerical aid. These results are consistent with a recent report [70] and suggest that the unique contribution of symbols to the emergence of numeracy may be to enhance the capacity for thinking about exact equality and correctly computing the effects of numerical transformations of sets.

We also did several follow up analyses to begin to test whether verbal numbers were a more effective symbolic tool compared to non-verbal symbols that rely on visuo-spatial supports in these initial stages of learning. While Western societies rely heavily on verbal-symbolic systems for counting and performing arithmetical computations, other cultures past and present have employed visuo-spatial symbolic supports (e.g., tally systems, abacus) for counting and calculation [see 86 for a review]. Over several analyses we did not see clear evidence that verbal-symbolic aids were significantly more effective than non-verbal symbolic aids, although more work is needed before this conclusion should be fully accepted.

Current theories diverge with regards to the particular combinations of core and cultural numerical abilities that are needed to drive conceptual development in number. These theories differ in nuanced and fundamental mechanistic ways that our current data do not have the precision to address. Furthermore, our analyses contrasting outcomes of verbal versus non-verbal symbolic training involved recombining training variants and, thus, were not orthogonal to other analyses. Nevertheless, our data bring some evidence to bear on one major division between theories: some implicate human language as the critical factor that combines with core number to produce conceptual change [e.g., 29, 39], while other theories propose that cultural-symbolic systems more broadly are the critical factor [87]. Exploratory analyses did not yield evidence of clear differences between those trained with verbal versus non-verbal symbolic aids on any of the numeracy assessments after training. To be clear, this is not evidence that verbal and non-verbal symbolic aids work equally well, nor is it strong evidence in favor of theories that emphasize symbolic systems broadly over language specifically. Nevertheless, these data are some of the first to contrast this distinction in an experimentally controlled manner and, at the least, suggest the possibility that symbols may not have to be verbal-linguistic in order to drive conceptual development in the emergence of numeracy.

To the extent that any differences were observed between the groups trained with verbal and nonverbal symbolic aids, they were isolated to pairwise comparisons on particular assessments. On the one hand, those trained with the non-verbal abacus counting performed better on the one-to-one correspondence task, which itself utilized an abacus, compared to those who received non-symbolic training. On the other hand, those trained with verbal symbolic aids performed better on the exact arithmetic transformation assessment task compared to those who received non-symbolic training. This finding aligns with previous evidence that count words may be essential to exact arithmetic [see 82]. This dissociation hints that verbal and non-verbal symbolic aids make differential contributions to conceptual development in the emergence of numeracy. Further pre-registered replication with larger samples, focused more specifically on this question, would be needed before drawing any strong conclusions.

Given our findings, there are at least three ways in which symbolic number aids might have led to reported improvements in non-verbal exact number tasks. It is possible that children who were trained with symbolic aids understood and/or learned numeracy concepts similarly to children trained without them, but those trained with symbolic aids were more likely to employ symbolic tools to solve these tasks after training even though the assessment tasks themselves did not demand the use of symbols. Such an account would accord with views that symbols are simply tools to extend resource limits for pre-existing human conceptual resources [88, 89]. Another possibility is that training with symbolic aids actually drove novel conceptual development [e.g., 29, 39]. If this is the case, symbolic number systems might provide the basis for children to conceive of exact equality and/or the effects of arithmetic transformation on exact number. Empirical work on the emergence of these concepts suggests that young children as well as people in cultures without formal symbolic number systems already have some conceptual resources for exact numerical representation through one-to-one correspondence [89, 90]. The effects of transformations (arithmetic or spatial/occlusion) on exact number, however, appear to be challenging early in development as well as for adults in cultures without symbolic number systems [89–92]. Thus, a third possibility is that symbols function differently for different aspects of early numeracy. For example, symbolic number may be a tool to extend existing, but limited, capacities for one-to-one correspondence [e.g., 89], paving the way for new conceptual developments regarding the effects of arithmetic transformations on exact number [e.g., 29, 39, 90].

In sum, this study tests the feasibility of using an experimental training framework to contrast specific hypotheses regarding the role of symbols in the emergence of numeracy as well as using numerical comparison in translational applications for improving numeracy. We find evidence that symbols uniquely aid understanding and performance related to one-to-one correspondence and exact arithmetic transformation. Our results also suggest that training with both verbal numbers and a non-verbal symbolic abacus led to gains in numeracy, but the contributions of each type of symbolic training may have been different. Our study provides the methodical and evidentiary groundwork to justify further tests of these and other ideas in a more fine-grained manner.

## Supporting information

S1 File This document includes all supporting information, including supporting tables (S1 and S2 Tables), figures (S1 –S3 Figs), and text (S1 and S2 Texts). (DOCX)
