# Supplementary material for: Testing the role of symbols in preschool numeracy: An experimental computer-based intervention study
Source: PLoS One. 2021 Nov 15;16(11):e0259775. doi: 10.1371/journal.pone.0259775 (PMC8592431; doi:10.1371/journal.pone.0259775)
Supplement: S1 File — (DOCX) [file pone.0259775.s001.docx]

**Supporting Information Document**

This document includes all supporting information, including supporting tables (Table S1, Table S2), figures (Fig S1, Fig S2, Fig S3), and text (Text S1, Text S2).

**Table S1. Major similarities and differences between training variants.**

| Condition | Comparison Type | Symbolic Number | Verbal Number | Presentation |
| --- | --- | --- | --- | --- |
| Sequential, Non-symbolic Enumeration | Approximate | No | No | Sequential |
| Set-based, Non-symbolic Enumeration | Exact | No | No | Sets |
| Abacus Counting | Exact | Yes | No | Sequential |
| Verbal Counting | Exact | Yes | Yes | Sequential |
| Verbal Labeling | Exact | Yes | Yes | Sets |

**Table S2. Number of participants contributing data for each training variant for each numeracy assessment task.** Cells include total number of participants from each training variant contributing both pre-test and post-test data for each numeracy assessment, as well as a breakdown by country-of-origin in the following format: TOTAL (U.S., Italy). Please note that due to resource constraints, the sequential, non-symbolic enumeration and the verbal labeling variants were run in the U.S. only.

|  | Counting Proficiency  (N = 215) | Numerical Comparison  (N = 211) | One-to-one correspondence.  (N = 161) | Arithmetic  Transformation  (N = 173) |
| --- | --- | --- | --- | --- |
| Sequential, Non-symb. | 21(21,0) | 21(21,0) | 20(20,0) | 20(20,0) |
| Set-Based, Non-symb. | 64(35,29) | 61(35,26) | 49(23,26) | 52(24,28) |
| Abacus Counting | 37(13,24) | 37(13,24) | 34(13,21) | 36(13,23) |
| Verbal Counting | 62(36,26) | 62(36,26) | 40(19,21) | 43(18,25) |
| Verbal Labeling | 31(31,0) | 30(30,0) | 18(18,0) | 22(22,0) |

**Fig S1. Flow diagram of data analysis inclusion.**

**
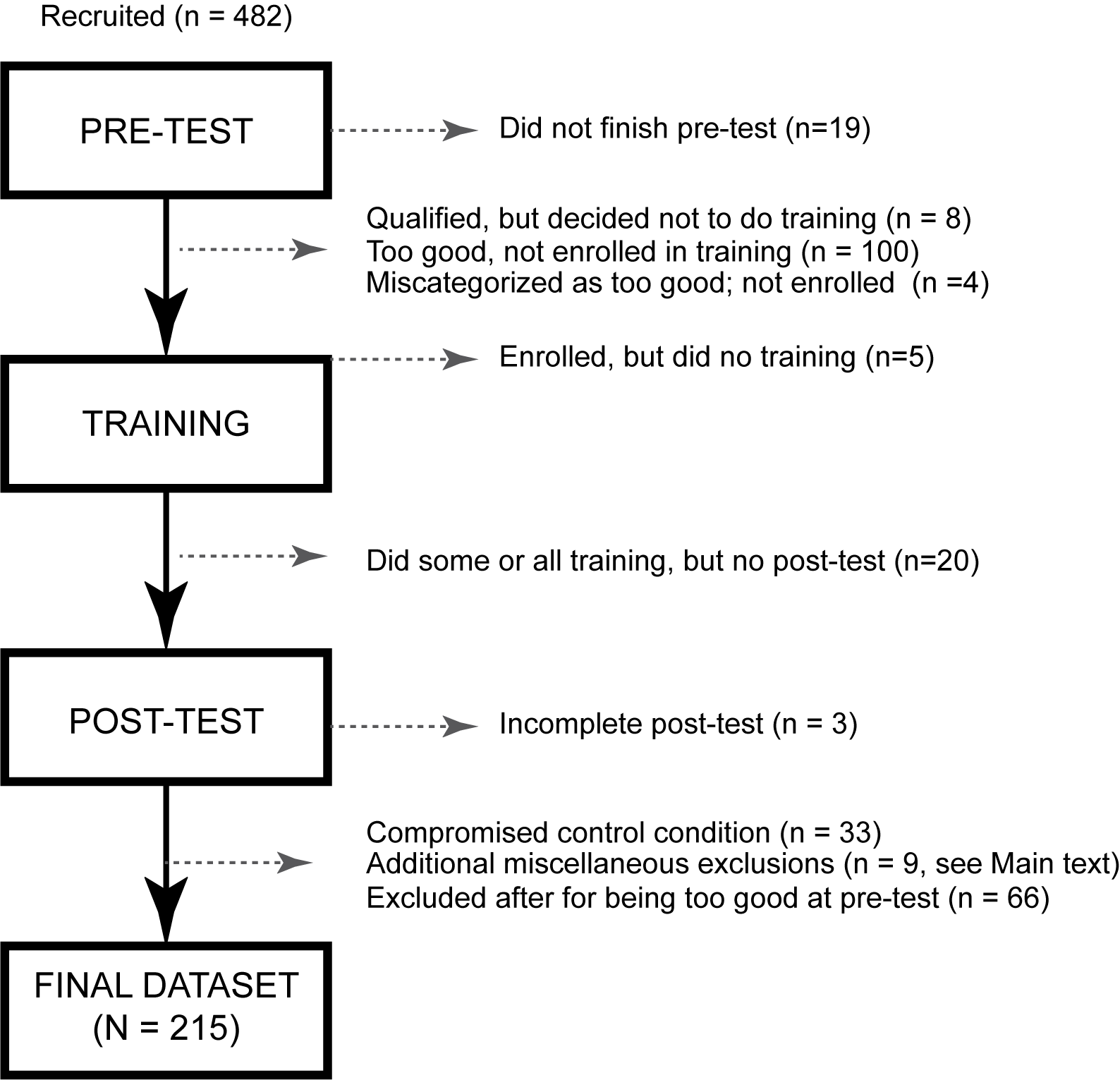
**

**Fig S2. Sensitivity power analysis curves for focused contrasts.** Power to detect different

sized effects as a function sample size.


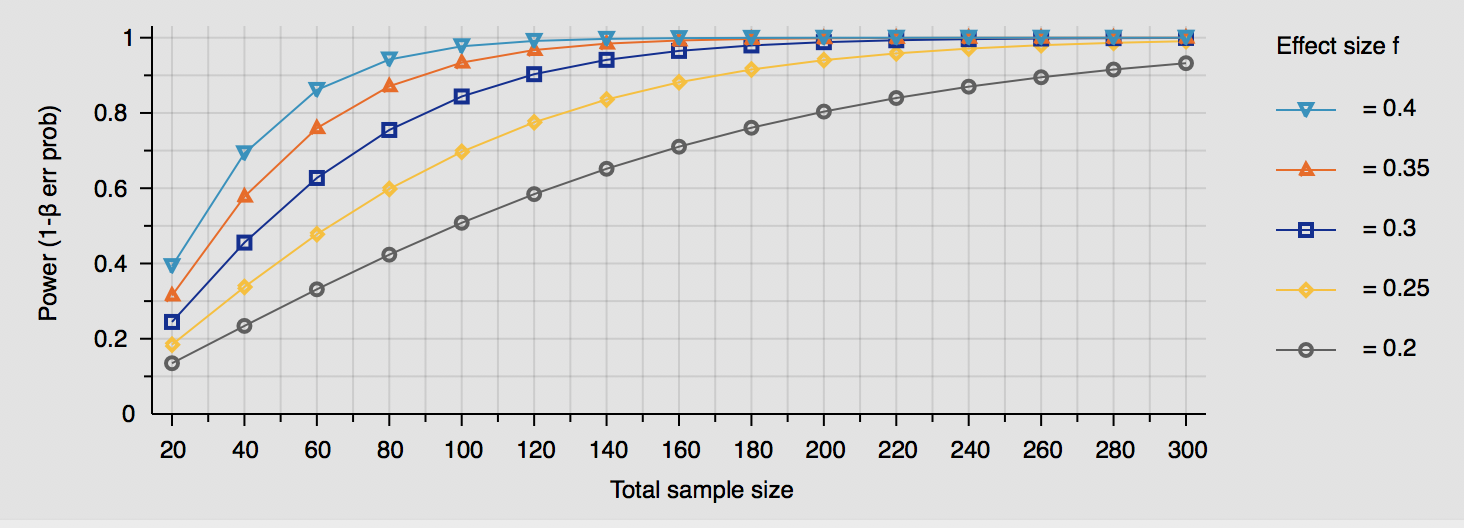


**Fig S3. Mean gains on assessments of numeracy after training for each of the five individual training variants.** Error bars are -/+ 1 SE.


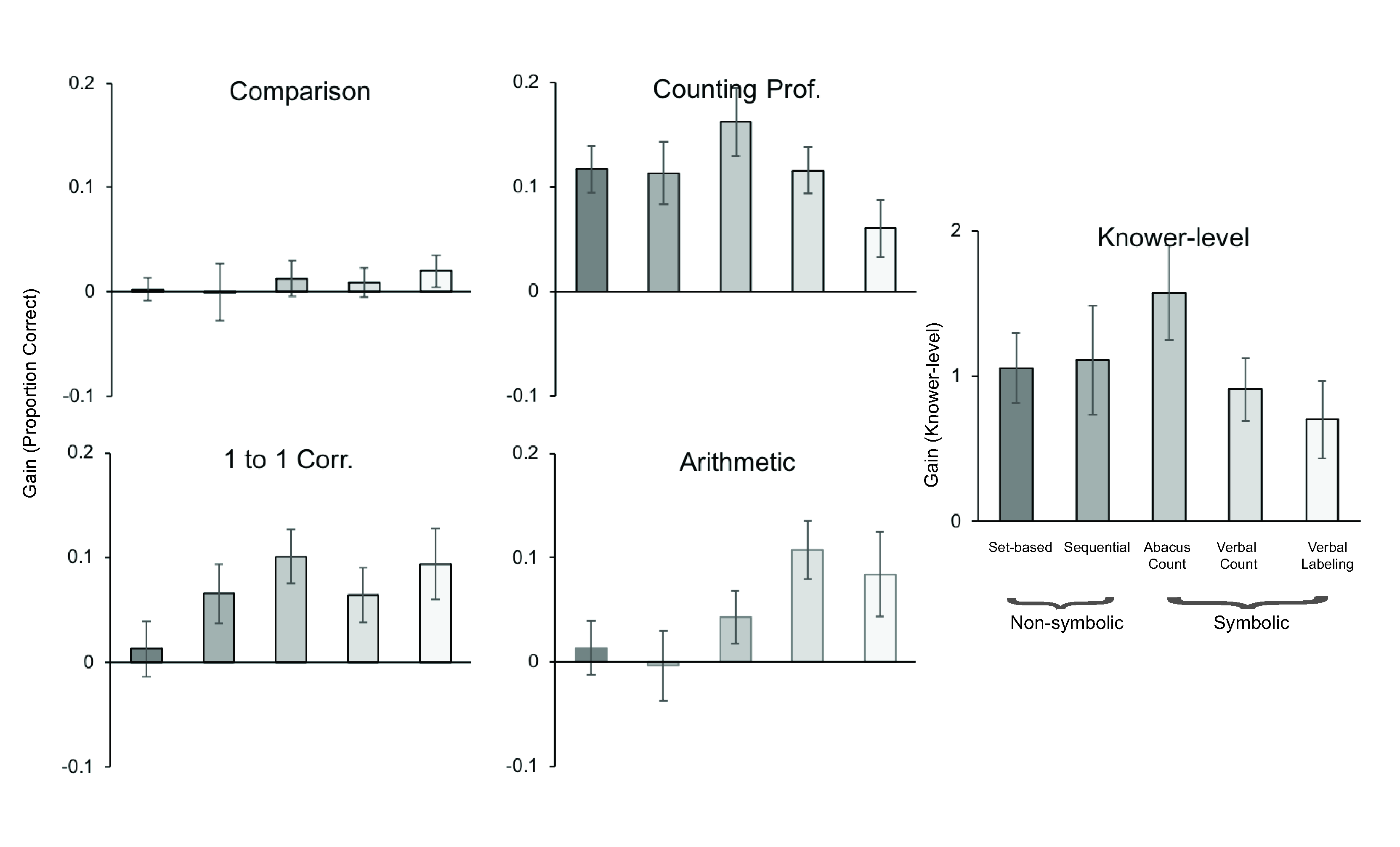


**Text S1. Pre-training abilities and training compliance between focused training variant groupings**

**Symbolic vs. non-symbolic**

Preliminary analyses showed that the symbolic (abacus counting, verbal counting, or verbal labeling, n =130) and non-symbolic (set-based or sequential non-symbolic enumeration, n = 85) variant groupings were well balanced before training. More specifically, there were no major differences between symbolic and non-symbolic training types in age (F(1,213) = 2.455, p = .119, partial eta squared = .011), count list knowledge (F(1,209) = 1.107, p = .294, partial eta squared = .005), or vocabulary (F(1,208) = 0.030, p = .862, partial eta squared < .001).

There were also no pre-test differences between variant groupings in counting proficiency (F (1,213) = 0.035, p = .852, partial eta squared < .001), numerical comparison (F (1,212) = 1.644, p = .201, partial eta squared = .008), arithmetic (F(1,175) = 0.827, p = .364, partial eta squared = .005), one-to-one correspondence (F(1,168) = 2.396, p = .124, partial eta squared = .014), or when counting proficiency was recoded into knower-levels (F(1,210) = 0.233, p = .630, partial eta squared = .001).

The symbolic and non-symbolic training groups were similarly compliant on the actual training. There were no major differences in the number of training trials completed (F(1,213) = 0.415, p = .520, partial eta squared = .002), proportion of correct answers in training (F(1,212) = 1.279, p = .259, partial eta squared = .006), or highest level of mastered (i.e., most difficult level reached over the course of training, F(1,204) = 0.012, p = .911, partial eta squared < .001) between these training types.

**Verbal vs. non-verbal**

Preliminary analyses showed that the recombined verbal (verbal counting or verbal labeling, n = 93) versus non-verbal (set-based enumeration, sequential non-symbolic enumeration, and abacus counting, n = 122) training variant groupings were also fairly well balanced at pretest. More specifically, there were no pretraining differences between verbal and non-verbal training types in age (F(1,213) = 0.793, p = .374, partial eta squared = .004), vocabulary (F(1,208) = 2.387, p = .124, partial eta squared = .011), or count list knowledge (F(1,209) = 0.026, p = .872, partial eta squared <.001).

There were also no pre-test differences between variant groupings in counting proficiency (F(1,213) = 0.303, p = .582, partial eta squared = .001), numerical comparison (F(1,212) = 0.002, p = .966, partial eta squared < .001), one-to-one correspondence (F(1,168) = 0.005, p = .942, partial eta squared < .001), or when counting proficiency was recoded into knower-levels (F(1,210) = 0.951, p = .331, partial eta squared = .005). The groupings did differ at pretest on arithmetic (F(1,175) = 7.662, p = .006, partial eta squared = .042), with those who received non-verbal numerical training having higher arithmetic scores before training than those that received verbal numerical training. As was standard in all our focused analyses, pre-test scores were always entered as a covariate in ANCOVA models to account for differences in starting point before training (see main text).

Verbal and non-verbal training groups also were similarly compliant on the actual training. There were no differences in number of training trials completed (F(1,213) = 0.306, p = .581, partial eta squared = .001), proportion of correct answers in training (F(1,212) = 0.559, p = .456, partial eta squared = .003), or level of mastery (F(1,204) = 0.653, p = .420, partial eta squared = .003) between training types.

**Symbolic verbal vs. symbolic non-verbal (Abacus)**

Preliminary analyses showed that there were no pretraining differences between non-verbal symbolic abacus (i.e., abacus counting, n = 37) and the verbal (verbal counting or verbal labeling, n = 93) training variant groupings in count list knowledge (F(1,126) = 0.643, p = .424, partial eta squared = .005). However, groups differed in age (F(1,128) = 7.412, p = .007, partial eta squared = .055) and vocabulary (F(1,125) = 4.522, p = .035, partial eta squared = .035) at pre-test. Significant effects between these variant groupings on training outcomes, if any, were followed up with additional analyses with age and vocabulary entered as covariates to account for these differences (see main text).

There were no pretraining differences between these variant groupings in counting proficiency (F(1,128) = 0.321, p = .572, partial eta squared = .003), numerical comparison (F(1,128) = 1.699, p = .195, partial eta squared = .013), one-to-one correspondence (F(1,97) = 1.484, p = .226, partial eta squared = .015), or when counting proficiency was recoded into knower-levels (F(1,126) = 0.765 p = .383, partial eta squared = .006).

Groups differed in pre-test arithmetic: F(1,101) = 7.298, p = .008, partial eta squared = .067), with those who received non-verbal symbolic numerical training being older and having higher vocabulary and arithmetic scores before training than those that received verbal symbolic training. As was standard in all our focused analyses, pre-test scores were always entered as a covariate in ANCOVA models to account for differences in starting point before training (see main text).

There were some differences on the actual training. The non-verbal symbolic training group completed had a higher proportion of correct answers in training than the verbal-symbolic group (F(1,128) = 4.120, p = .044, partial eta squared = .031). There were no differences in number of training trials completed (F(1,128) = 1.998, p = .160, partial eta squared = .015),or highest level of mastery (F(1,122) = 1.555, p = .215, partial eta squared = .013).

**Abacus vs. non-symbolic**

There were no pretest differences between training with the non-verbal symbolic abacus (n = 37) and those who trained with non-symbolic numerical comparison only (set-based or sequential non-symbolic enumeration, n = 85) on vocabulary (F(1,116) = 1.613, p = .207, partial eta squared = .014), count list knowledge (F(1,117) = 1.679, p = .198, partial eta squared = .014). However, there was a grouping difference by age (F(1,120) = 8.639, p = .004, partial eta squared = .067). Significant effects between these variant groupings on training outcomes, if any, were followed up with additional analyses with age entered as a covariate to account for these differences.

There were no pre-test differences between these training groupings in counting proficiency (F(1,120) = 0.086, p = .770, partial eta squared = .001), numerical comparison (F(1,119) = 3.375, p = .069, partial eta squared = .028), arithmetic (F(1,109) = 1.296, p = .257, partial eta squared = .012), one-to-one correspondence (F(1,104) = 3.589, p = .061, partial eta squared = .033), or when counting proficiency was recoded into knower-levels (F(1,118)= 0.086, p = .770, partial eta squared = .001).

There was no difference in total training trials completed (F(1,120) = 2.029, p = .157, partial eta squared = .017) or highest training level reached (F(1,117) = 0.801, p = .373, partial eta squared = .007). However, there was a difference between groupings in proportion of correct answers in training (F(1,119) = 6.152, p = .015, partial eta squared = .049).

**Verbal vs. non-symbolic**

There were no pretest differences between those who trained with verbal symbolic aids (verbal counting or verbal labeling, n = 93) and those who trained with non-symbolic numerical comparison (set-based or sequential non-symbolic enumeration, n = 85) on age (F(1,176) = 0.219, p = .640, partial eta squared =.001), vocabulary (F(1,173) = 0.806, p = .371, partial eta squared = .005), or count list knowledge (F(1,173) = 0.497, p = .482, partial eta squared =.003).

There were also no differences between these groupings in pre-test counting proficiency (F(1,176) = 0.157, p = .693, partial eta squared = .001), numerical comparison (F(1,175) = 0.520, p = .472, partial eta squared = .003), one-to-one correspondence (F(1,133) = 0.847, p = .359, partial eta squared = .006), or when counting proficiency was recoded into knower-levels (F(1,174)= 0.627, p = .429, partial eta squared = .004). However, there was a pre-test difference in arithmetic (F(1,138) = 4.967, p = .027, partial eta squared =.035). As in all of our focused analyses, pre-test score was entered as a covariate to account for these differences.

There were no differences in total training trials completed (F(1,176) =0.006, p = .939, partial eta squared < .001), highest training level reached during training (F(1,167) = 0.140, p = .708, partial eta squared = .001), or in the proportion of correct answers in training (F(1,175) = 0.068, p = .794, partial eta squared < .001) between these groupings.

**Text S2. Sensitivity Power Analysis**

We conducted sensitivity power analyses for each of the main focused analyses in G*Power 3 (statistical design settings: test family = of F tests; Statistical test= ANCOVA: fixed effects, main effects and interactions; Numerator df = 1, Number of groups = 2; Number of covariates = 1). Fig S2 shows sensitivity curves for different numbers of subjects and different effect sizes, given our statistical design. Overall, the sensitivity analyses revealed that we should be able to detect at least medium effect sizes ranging from Cohen’s *f* = .2470-.3800 (Cohen, 1969) with a high degree of power (.95) at our desired alpha level (p = .05) with our statistical design (ANCOVA comparing 2 groups with 1 covariate) and the total number of subjects included in our contrasts (n = 92-215).

For outcome contrasts of those trained on symbolic variants to those trained on non-symbolic variants specifically, the analyses suggested we could reasonably detect medium effect sizes between *f* = .2470-.2858 with a high degree of power (.95) with the obtained total sample sizes (n = 161-215).

For outcome contrasts between those trained on variants with verbal numbers to those trained on other all variants without verbal numbers, the analyses similarly suggested that we could reasonably detect medium effect sizes between *f* = .2470-.2858 with a high degree of power (.95) with the obtained total sample sizes (n = 161-215).

For outcome contrasts between those trained on variants with symbolic verbal number aids to those trained on the variant with the symbolic abacus aid, the analyses suggested that we could reasonably detect medium effect sizes between f = .3186-.3800 with a high degree of power (.95) with the obtained total sample sizes (n =92-130).

Finally, the analyses suggested we could reasonably detect medium effect sizes for contrasts of verbal-symbolic training outcomes to non-symbolic training outcomes (n =127-137, *f =* .3102-.3224) and for contrasts of abacus training outcomes to non-symbolic training outcomes (n = 103-108, *f* = .3501-.3587) with our obtained samples sizes.
